# The role of intelligence in PTSD treatment outcomes: evidence from a multicenter TF-CBT trial with youth

**DOI:** 10.1186/s13034-026-01149-7

**Published:** 2026-07-31

**Authors:** Sabrina Berardi, Hanna Christiansen, Sebastian Deutscher, David Daniel Ebert, Rebekka Eilers, Katharina Gossmann, Anne Grass, Lena Jaworski, Johanna Kneidinger, Rita Rosner, Katharina Szota, Anna-Carlotta Zarski, Regina Steil

**Affiliations:** 1 Department of Clinical Psychology and Psychotherapy, Institute of Psychology, Zentrum Für Psychotherapie (ZPT)/Center for Psychotherapy, Goethe University Frankfurt, Frankfurt am Main, Germany; 2 Department of Psychology, Catholic University Eichstätt-Ingolstadt, Eichstätt, Germany; 3 Institute for Clinical Child and Adolescent Psychology, Deutsches Zentrum Für Psychische Gesundheit (DZPG)/German Center for Mental Health, Philipps University Marburg, Marburg, Germany; 4 Department of Sport and Health Sciences, School of Medicine and Health, Technical University Munich, Munich, Germany; 5 Division of eHealth in Clinical Psychology, Department of Clinical Psychology, Philipps University Marburg, Marburg, Germany; 6 Dynamic Center, LOEWE1/16/519/03/09.001(0009)/98, Frankfurt am Main, Germany

**Keywords:** Posttraumatic stress disorder, Trauma-focused cognitive behavioral therapy, Intelligence, Therapy outcome, Dropout

## Abstract

**Introduction:**

Cognitive ability has been linked to posttraumatic stress disorder (PTSD) vulnerability and may influence trauma-focused treatment response, yet its role in youth Trauma-Focused Cognitive Behavioral Therapy (TF-CBT) outcomes remains unclear. Clarifying this issue is relevant for equitable access to evidence-based care. We examined whether formally assessed IQ was associated with PTSD symptom change from pre- to post-treatment based on youth and caregiver reports and with treatment dropout in children and adolescents undergoing TF-CBT after abuse or neglect.

**Method:**

Data came from *N* = 320 youth aged 5–21 years from a randomized clinical effectiveness trial. Intelligence quotient (IQ) was assessed with standardized tests, PTSD symptoms with the Child and Adolescent Trauma Screen (CATS-2), and dropout was defined as not completing therapy as intended. Symptom change was calculated as post- (T1) minus pre-treatment (T0) and evaluated separately for youth and caregiver reports. Regression models included age, gender, baseline PTSD severity, and treatment satisfaction assessed after treatment as an additional model variable.

**Results:**

Mean IQ was *M* = 97.41 (*SD* = 13.19, range = 51–138). PTSD severity was high at baseline (*M* = 34.21, *SD* = 8.09) and decreased substantially (ΔCATS-2_T1 ₋ T0_ youth report: *M* = −17.54, *SD* = 12.34; Cohen’s *d* paired = 1.42). IQ showed small but significant negative correlations with symptom change in youth reports (*r* = −.14, *p* = .040) and caregiver reports (*r* = −.17, *p* = .018), indicating greater symptom reductions at higher IQ. In regressions, IQ was not a significant predictor in the youth-report model (β = −.10, *p* = .077), while a small effect remained in the caregiver-report model (β = −.15, *p* = .011). Baseline PTSD severity and post-treatment satisfaction showed more consistent associations with symptom reduction. Dropout was 29.1%. IQ neither differed between completers and dropouts nor was associated with dropout in logistic regression (OR = 0.99, *p* = .536), whereas higher post-treatment satisfaction was associated with lower dropout risk.

**Discussion:**

TF-CBT was associated with substantial symptom reductions across the observed IQ range, suggesting its suitability for routine care without excluding youth solely on the basis of cognitive ability. Baseline severity and treatment satisfaction at post-treatment showed stronger associations with symptom change and dropout than IQ.

*Trial registration* The BESTFORCAN study was registered in the German Clinical Trial Registry (DRKS00020516) on 12 February 2020.

## Introduction

Traumatic experiences are common in childhood and adolescence, with meta-analytic estimates indicating that approximately 15–20% of trauma-exposed youth develop post-traumatic stress disorder (PTSD) [2, 81]. Lower cognitive ability has been linked to a greater likelihood of trauma exposure [5, 34] and to an increased risk of victimization, including substantially higher maltreatment rates among children with intellectual disabilities than among their typically developing peers [3, 10]. Among trauma-exposed individuals, lower cognitive ability has also been associated with a greater risk of PTSD [4, 33, 36, 64], consistent with meta-analytic and review evidence identifying lower cognitive ability as a pre-existing vulnerability factor for the disorder [6, 46, 55]. Prospective studies in U.S. veterans found that lower pre-deployment intelligence predicted greater PTSD risk [35] and symptom severity [38]. More recently, Zalmenson et al. [87] followed 582 soldiers across multiple deployments and found that lower general intelligence quotient (IQ), particularly lower abstract reasoning, predicted steeper increases in PTSD symptoms over time, with this association partly mediated by combat exposure. However, not all findings are consistent. Shura et al. [66] reported that apparent IQ–PTSD associations in 338 veterans largely disappeared after accounting for performance and symptom validity checks, suggesting that some prior estimates may be methodologically inflated.

In youth, longitudinal research by Breslau et al. [5] found that children with above-average IQ scores (> 115 at age 6) were less likely to experience traumatic events by age 17 and, if exposed, had a lower likelihood of developing PTSD compared to peers with lower IQ scores. One explanation is that children with higher IQ may deploy more effective cognitive strategies, including better risk recognition, personal safety awareness, adaptive coping, and problem solving, which may act protectively both before and after trauma [36, 47].

Yet the relationship between intelligence and PTSD is not unidirectional. Trauma exposure may itself compromise cognitive development, particularly during sensitive periods of brain maturation, and thereby increase vulnerability to further adversity and psychological difficulties [21, 30, 31, 65]. For example, Kira, Lewandowski, Somers, et al. [30, 31] found that among 390 highly traumatized adolescents, cumulative trauma was negatively associated with perceptual reasoning, working memory, processing speed, and verbal comprehension, with PTSD symptoms partially mediating these associations. Further distinguishing between trauma exposure and PTSD, Saigh et al. [63] compared IQ scores of traumatized youth with and without PTSD and found that PTSD, rather than trauma exposure per se, was specifically associated with lower verbal IQ. Cross-diagnostic evidence further supports the interplay between childhood maltreatment, subclinical post-traumatic stress symptoms, and IQ [67]. For instance, Delaney-Black et al. [18] found that violence-exposed inner-city children scored significantly lower on standardized IQ and reading tests than non-exposed peers, even after adjustment for socioeconomic and familial variables. At a broader level, a meta-analysis of 55 studies found small-to-medium deficits in working memory, inhibition, and cognitive flexibility in trauma-exposed youth relative to non-exposed peers ([53]). Neuroimaging studies may provide a neurobiological context for these findings, linking early adversity to structural changes in the prefrontal cortex, hippocampus, and amygdala, regions involved in executive and emotional functioning [45, 76]. Danese et al. [11] emphasize the importance of assessing cognitive functioning prior to trauma exposure to clarify causal pathways. Their longitudinal data suggest that lower cognitive ability may both increase the risk of victimization and contribute to later PTSD vulnerability, indicating that cognitive factors can be both antecedents and consequences of trauma.

Given that cognitive ability both shapes and is shaped by trauma, a natural question is whether it also influences treatment response. Regarding PTSD, earlier research suggested that cognitive abilities such as verbal memory and reasoning may influence treatment response [84]. Marx et al. [40] found that adults with higher clinically assessed IQ showed greater early PTSD symptom reductions during *Cognitive Processing Therapy* (CPT; [57]), although these effects were not consistently maintained at follow-up and educational attainment, used as a proxy, did not moderate outcomes. In contrast, Rizvi et al. [58] reported no significant relationship between intelligence and PTSD treatment outcomes in women with assault-related PTSD undergoing CPT, possibly because of restricted variability in cognitive ability. In a naturalistic study of veterans with PTSD and comorbid mild traumatic brain injury, Tanev et al. [75] found that neither pretreatment cognitive performance nor cognitive impairment predicted response to cognitive-behavioral therapies, and therefore discouraged excluding patients with lower cognitive ability from these treatments.

While findings in adults regarding the association between intelligence and PTSD treatment response are mixed, corresponding research in children and adolescents remains scarce. Existing studies in youth samples have often relied on proxy indicators such as educational attainment rather than standardized IQ tests [17, 72], and many are limited by small sample sizes and restricted variability in cognitive ability, reducing the robustness and generalizability of findings. A recent exception is Steil et al. [71], who assessed IQ in 44 adolescents and young adults (aged 14–21) with abuse-related PTSD who received developmentally adapted CPT and found no association between IQ and PTSD symptom reduction, remission, or dropout—though the small sample limits statistical power. In one of the few other youth-specific studies, Mathiassen et al. [41] found that higher full-scale IQ predicted steeper symptom improvement in a child and adolescent outpatient mental health sample, suggesting that cognitive ability may function as a moderator of psychotherapy outcome in young populations, although this finding was not specific to trauma-focused treatment.

Beyond methodological limitations, the scarcity of youth-specific research is particularly consequential from a developmental perspective. Cognitive abilities in children and adolescents are still maturing, and trauma-related disruptions—including heightened stress reactivity, dysregulated affect, and reduced school engagement—may further compromise cognitive functioning during this critical period [18, 44]. Findings from adult samples therefore cannot simply be extrapolated to youth, underscoring the need for rigorous, developmentally sensitive research using direct IQ measures to clarify the role of cognitive ability in youth PTSD treatment outcomes.

This gap is particularly relevant for *Trauma-Focused Cognitive Behavioral Therapy* (TF-CBT; [8]), the most empirically supported intervention for PTSD in children and adolescents and a first-line treatment in international guidelines [ 51]. Unlike approaches originally developed for adults and subsequently adapted for younger populations, TF-CBT was designed from the outset for children and adolescents. Its structured components, including psychoeducation, affect regulation, cognitive coping, and the trauma narrative, are intended to be flexibly adapted to different developmental stages and cognitive levels [8]. At the same time, TF-CBT's reliance on cognitive restructuring, narrative processing, and caregiver involvement makes it a treatment in which cognitive ability could plausibly influence engagement and outcomes.

Beyond its potential influence on symptom improvement, cognitive ability may also affect treatment retention. Treatment dropout constitutes a central indicator of therapy outcome, as premature termination limits the effectiveness and dissemination of evidence-based interventions [73]. Meta-analyses in adults indicate substantial variability in dropout rates from trauma-focused treatments, depending on the population, treatment setting, and study design [27, 37, 80, 86]. Evidence from youth samples is mixed, with some meta-analyses reporting relatively low and others considerably higher dropout rates [69, 83], underscoring the need to better understand the factors that predict dropout. Previous research has linked symptom severity, comorbidity, age, and therapeutic alliance to dropout [27, 37, 49, 54, 83, 88]. Cognitive characteristics such as intelligence have received little attention as predictors of dropout, although O’Keeffe et al. [52] found that lower verbal intelligence predicted premature termination in depressed adolescents receiving psychotherapy, alongside missed sessions and poor therapeutic alliance. In younger children referred for oppositional and antisocial behavior, Kazdin and Mazurick [28] similarly found that lower IQ predicted premature termination from treatment, suggesting that cognitive ability may play a role in treatment retention across different clinical presentations and age groups. In adult PTSD samples, Tillman et al. [78] reported that higher IQ was associated with greater treatment completion rates in CPT. Whether intelligence is associated with treatment retention in youth receiving trauma-focused treatment remains unknown.

Given its central role in treating PTSD in children and adolescents, TF-CBT provides a particularly relevant context for examining the role of cognitive ability in therapy. However, associations between IQ and symptom change or treatment retention have not yet been investigated in TF-CBT. This lack of evidence is clinically important, as youth with lower cognitive functioning are particularly vulnerable yet should not be excluded from evidence-based care, raising questions of equitable access. If lower IQ limited treatment response, clinicians would need to adapt TF-CBT materials, techniques, and pacing to ensure accessibility for all patients. Conversely, if TF-CBT proves equally effective across a wide range of cognitive abilities, this would support its broad applicability in routine care and large-scale dissemination without major modifications. Clarifying the role of cognitive ability in TF-CBT is therefore essential both for advancing theory on cognitive moderators of treatment response and for guiding equitable and effective clinical practice.

The present study addresses this gap by investigating whether standardized IQ scores are associated with (I) reductions in PTSD symptom severity from pre- to post-treatment, based on both self- and caregiver reports, and (II) the likelihood of treatment dropout in a large multicenter dissemination trial in Germany involving youth who had experienced abuse or neglect. Given the limited and inconsistent previous findings, we did not formulate directional hypotheses.

To examine the association between IQ and treatment outcomes while accounting for relevant demographic and clinical factors, analyses included age, gender, and baseline PTSD symptom severity, which have previously been linked to symptom change in trauma-focused treatment for youth [12, 14]. Treatment satisfaction assessed after treatment was included as an additional model variable because perceived treatment experience may be associated with clinical change in youth mental health care [23, 24].

## Method

We used data from the BESTFORCAN study, a multicenter, cluster-randomized controlled trial (RCT) that examined the implementation of TF-CBT for children and adolescents with posttraumatic stress symptoms (PTSS) after child abuse and neglect (CAN). This dissemination study evaluated the effectiveness of specialized trauma-focused supervision compared to supervision as usual (German Clinical Trials Register identifier: DRKS00020516). The study was approved by the ethics committees of the Universities Frankfurt am Main, Eichstätt-Ingolstadt, Erlangen and Marburg. Details of the study design are described elsewhere [59, 60].

### Procedure and participants

To be eligible for the study, participants had to be children or adolescents between the ages of 5 and 21 who had experienced CAN according to the event criteria proposed for ICD-11 [68, 70]. Participants were required to meet diagnostic criteria for maltreatment-related PTSD according to ICD-10, or to have any mental disorder and score at least 21 points on the *Child and Adolescent Trauma Screen version 2* (CATS-2; [61]). Originally, at least one caregiver had to be available and willing to regularly participate in treatment sessions. This requirement was later adjusted to better accommodate practical considerations as some participants lacked stable caregiving arrangements, and older adolescents demonstrated sufficient autonomy to engage in treatment independently. The exclusion criteria were set to ensure that participants could safely and effectively engage in TF-CBT. Excluded from participation were those with an IQ below 50, those with current psychosis, severe substance dependence, acute suicidality, or those receiving concurrent psychotherapy. The use of stable psychotropic medication was permitted. The study also required that participants were in a stable environment, with no ongoing abuse or neglect occurring during the study period.

Therapists (either licensed psychotherapists [LPTs] or psychotherapists in training [PITs]) were randomized into two groups: trauma-focused supervision (TFS) or supervision as usual (SAU). The TFS group received bi-weekly consultations with trained supervisors to ensure adherence to the TF-CBT manual, whereas the SAU group engaged in supervision as usual in their professional training programs or their practices. Patients were naturally assigned to therapists based on referral paths and availability.

### Treatment

*TF-CBT* [8] is a manualized therapy designed to treat PTSS in children and adolescents. The therapy typically consists of 12 to 25 sessions, depending on clinical need, and integrates caregivers into the treatment process to provide additional support. Treatment is typically delivered in 100-min double sessions, with one half conducted with the child and the other with the caregiver. In the present study, therapists were able to adapt this format to single sessions when double sessions were not feasible in routine care. The therapy follows a structured, step-by-step approach, covering key components in nine core modules: (1) Psychoeducation, (2) Parenting Skills, (3) Relaxation, (4) Affect Regulation, (5) Cognitive Coping, (6) Trauma Narrative Development, (7) In Vivo Exposure, (8) Joint child-caregiver sessions on Problem-Solving, and (9) Enhancing Safety and Developmental Skills. Each module is designed to build specific competencies in both the child and the caregiver to help manage trauma-related symptoms. A central feature of TF-CBT is the trauma narrative, in which the child progressively recounts the traumatic experience in collaboration with the therapist. This narrative can take various forms, such as writing, drawing, or symbolic expression, depending on the child's age and developmental stage. Once completed, the narrative is typically shared with the caregiver during a joint session (when available), supporting emotional processing of the traumatic experience and strengthening the caregiving environment. Sessions preceding the trauma narrative focus on psychoeducation and equipping both the child and caregiver with coping skills, whereas subsequent sessions address cognitive restructuring and difficulties that may persist following exposure, while promoting future development. This structured and adaptable framework ensures that TF-CBT can be effectively integrated into routine care, while the main study aimed to assess how the type and quality of supervision impact treatment outcomes for children with trauma-related symptoms.

### Measures

Assessments were conducted at baseline (T0, pre-treatment) and post-treatment (T1). At baseline,* demographic information* was collected, including age, gender, residency (e.g., in care), school attendance, and socioeconomic background. These data were obtained through self- and caregiver-reports as well as therapist documentation.

*IQ.* General cognitive functioning was assessed at baseline using standardized, age-appropriate intelligence tests from therapists’ routine diagnostic repertoire, in accordance with standard German clinical practice for youth mental health diagnostics (e.g., *Culture Fair Intelligence Test, Scale 2—Revised* [CFT 20-R]; *Wechsler Intelligence Scale for Children—Fifth Edition* [WISC-V]). In line with the naturalistic dissemination design, the specific IQ measure used varied across sites and was selected according to the participant’s age, clinical context, prior diagnostic information, and local routine practice. All instruments were validated and normed for the relevant age groups. If available, IQ scores could also be obtained from prior assessments conducted by other clinicians, provided that standardized intelligence measures had been used. IQ scores were documented for each participant by their therapist. Thus, IQ scores were treated as broad clinical estimates rather than as measures derived from one uniform intelligence test.

*PTSS.* PTSS were assessed using the German adaptation of the *Child and Adolescent Trauma Screen* (CATS-2; [62]), a validated instrument aligned with DSM-5 and ICD-11 criteria. The CATS-2 comprises three sections: a trauma exposure checklist, 20 symptom items, and a functional impairment scale. Symptom items refer to the past four weeks and cover the DSM-5 clusters of intrusion, avoidance, negative alterations in cognitions and mood, and alterations in arousal. Responses are rated on a 4-point Likert-type scale ranging from 0 (“never”) to 3 (“almost always”), with higher scores indicating greater symptom severity. For analyses, we used the DSM-5 PTSD symptom sum score (0–60) from items 1–20, scoring multi-part items using the highest endorsed subitem once (per CATS-2 manual). For interpretation, Sachser et al. [62] reported that a CATS-2 DSM-5 PTSD score of ≥ 21 is a sensitive cut-off optimal for screening purposes, whereas a score of ≥ 25 shows higher specificity for probable PTSD diagnoses. The CATS-2 was administered at pre-treatment (T0) and post-treatment (T1). Both the child/adolescent and the caregiver completed the instrument independently at each time point. Baseline assessments were supervised by trained assessors. Internal consistency at T0 for the CATS-2 DSM-5 symptom scale was α = 0.84 (self-report) and α = 0.89 (caregiver-report), which is comparable to the original validation (α = 0.89/0.91; [62]).

*Treatment Satisfaction*. Treatment satisfaction was assessed using the *Fragebogen zur Beurteilung der Behandlung* (FBB; *Treatment Assessment Questionnaire;* [42]), a validated feedback instrument available in versions for children and adolescents (FBB-KJ) and caregivers (FBB-E). The adolescent version comprises 20 scored items across three domains: treatment outcome (5 items), therapeutic relationship (7 items), and treatment setting and conditions (8 items), plus one open-ended prompt. The caregiver version comprises 21 scored items across two domains: treatment outcome (7 items) and treatment process and course (13 items), plus a single global satisfaction item and one open-ended prompt. Items are rated on a 0–4 scale (0 = “not at all/never” [„überhaupt nicht/niemals“], 4 = “completely/always” [„ganz genau/immer “]); specified items are reverse-coded, and subscale and total scores are computed as item means, with higher values indicating greater satisfaction. In the present study, overall treatment satisfaction was operationalized as the total score (item mean) across all scored items of each version (excluding the global item and open-ended prompts). Both versions were administered post-treatment (T1) and completed independently by the respective informants. Internal consistency at T1 for the total score was α = 0.88 (caregiver-report), indicating good reliability, consistent with the values reported in the test manual (α > 0.80 across versions; [42]), and α = 0.54 (child-report), indicating low internal consistency in the present sample. The lower alpha in the child-report may reflect the multidimensional structure of the instrument and developmental variability in children’s interpretations of satisfaction-related items. Findings involving this variable should therefore be interpreted with caution. In addition, because satisfaction was assessed after treatment, it cannot be interpreted as a baseline or prospective predictor of symptom change or dropout.

*Dropout*. The dataset distinguished between child-/family-initiated and therapist-initiated premature terminations. In some cases, both codes were marked (e.g., when termination was mutually agreed upon after initial dropout intentions). For the purposes of this analysis, we defined dropout as any case where the child-/family-initiated code was marked, regardless of therapist involvement. This operationalization ensured that dropout reflected a discontinuation not primarily initiated by the therapist alone.

### Data analysis

To address the methodological limitations of previous studies and test our non-directional hypotheses, we conducted a series of inferential analyses. For Hypothesis 1, we used Pearson’s correlation coefficients to examine the relationship between intelligence (IQ) and PTSD symptom reduction from T0 to T1 assessment. Symptom change was operationalized as the difference between post- and pre-treatment CATS-2 sum scores (ΔCATS-2 = CATS-2_T1_ − CATS-2_T0_), separately for child self-report (ΔCATS-2_CHILD) and caregiver report (ΔCATS-2_CARE). Subsequently, multiple linear regression analyses were performed to examine whether IQ was associated with symptom change while adjusting for age, gender, baseline PTSD severity, and treatment satisfaction assessed after treatment. For Hypothesis 2, an independent-samples *t* test was conducted to compare IQ scores between participants who completed treatment and those who dropped out. In addition, a binary logistic regression was performed to examine whether IQ was associated with dropout likelihood while adjusting for the same model variables.

The analyses were exploratory and based on non-directional hypotheses. Age, gender, and baseline PTSD severity were included as baseline covariates. Because treatment satisfaction was assessed after treatment, it was not interpreted as a baseline or prospective predictor. Instead, it was included as an additional model variable to examine whether perceived treatment experience was associated with symptom change and dropout in the adjusted models.

Participants who self-identified their gender as “diverse” (*n* = 14) were excluded from regression analyses, as treating this small group as a separate category would have resulted in unstable parameter estimates and limited interpretability of effects. Accordingly, sample sizes for regression models were slightly smaller than for correlational analyses; however, descriptive statistics and correlations are reported for the full sample.

Statistical assumptions were examined, and diagnostics for normality, linearity, homoscedasticity, and multicollinearity indicated that the assumptions were sufficiently met. Given the large sample size and the robustness of the applied procedures [22, 74], all analyses were conducted with untransformed data. Statistical analyses were conducted using IBM SPSS Statistics (Version 29.0.2.0 for Windows) with α set at 0.05.

## Results

### Sample characteristics

The initial sample comprised *N* = 376 participants. After excluding cases with missing standardized IQ or baseline assessments, the final subsample included *n* = 320 youth (69.7% female, 25.9% male, 4.4% diverse). Participants had a mean age of *M* = 14.48 years (*SD* = 3.58, range = 5–21) and were mostly enrolled in grades 8–9 across various school types. The primary diagnosis was PTSD (F43.1; 83.1%), followed by other stress-related disorders (F43.x; 7.2%). Frequent comorbidities included depressive disorders (17.2%) and social phobia (5.3%). The mean IQ was *M* = 97.41 (*SD* = 13.19, range = 51–138).

Of 320 documented caregivers, *n* = 293 provided valid information on their relationship to the child. Parents represented the largest group (61.1%). Approximately a quarter were professional caregivers or residential staff (23.2%), with the remainder comprising foster parents (5.1%), grandparents (2.0%), other relatives (3.1%), and non-relatives (3.4%). Caregivers had a mean age of *M* = 42.10 years (*SD* = 10.63, range = 12–77). Among those reporting gender (*n* = 295), 82.7% were female, 16.9% male, and 0.3% diverse. Educational data (*n* = 243) showed that 16.0% had completed lower secondary (*Haupt-/Volksschule*), 40.3% obtained an intermediate secondary qualification (*Realschule/Mittlere Reife/Polytechnische Oberschule*), and 39.1% completed upper secondary with a general university entrance qualification (*Abitur*). In terms of current or most recent primary employment (*n* = 225 valid), 85.3% were employees, 7.1% were self-employed (7.6% including farmers), 2.7% were civil servants, and 3.1% had never been employed. On average, households reported €3,132 in monthly net income (*SD* = €2,547; *n* = 199) and had 3.61 members (*n* = 240). Overall, the caregiver sample was diverse in familial role, demographic characteristics, and socioeconomic background, reflecting the heterogeneous care contexts in which TF-CBT was implemented.

### Descriptive data

At pre-treatment (T0), the mean self-reported PTSD symptom score (CATS-2_CHILD) was *M* = 34.21 (*SD* = 8.09, *n* = 320). At post-treatment (T1), the mean CATS-2_CHILD score was *M* = 16.63 (*SD* = 11.41, *n* = 226). Symptom change was calculated as the difference between post- and pre-treatment scores (ΔCATS-2_CHILD = CATS-2_CHILD_T1_ − CATS-2_CHILD_T0_). The mean change was *M* = −17.54 (*SD* = 12.34, *n* = 226, range = −49 to 25), corresponding to a large effect size, Cohen’s *d* paired = 1.42. Caregiver-reported PTSS scores (CATS-2_CARE) were *M* = 29.53 (*SD* = 10.03, *n* = 300) at T0 and *M* = 16.10 (*SD* = 10.31, *n* = 208) at T1. Symptom change was calculated analogously (ΔCATS-2_CARE = CATS-2_CARE_T1_ − CATS-2_CARE_T0_​). The mean change was *M* = −12.88 (*SD* = 11.69, *n* = 205, range = −45 to 20), corresponding to a large effect size, Cohen’s *d* paired = 1.10. Overall treatment satisfaction was high, with youth reporting a mean overall satisfaction score of *M* = 3.32 (*SD* = 0.49, *n* = 226, range = 1.30–4.00) and caregivers reporting *M* = 3.58 (*SD* = 0.40, *n* = 208, range = 1.81–4.00). To further characterize the sample, completers (*n* = 227, 70.9%) and dropouts (*n* = 93, 29.1%) were compared on baseline characteristics (see Table 1). Groups were similar in IQ and child-reported PTSD symptoms. However, dropouts showed higher caregiver-reported PTSD severity at baseline and markedly lower treatment satisfaction at post-treatment in both child and caregiver reports.

**Table 1:** Descriptive statistics for completers and dropouts and group comparisons

Variable	Completers *M* (*SD*) [*n*], Min–Max	Dropouts *M* (*SD*) [*n*], Min–Max	*t*(df)	*p*
IQ	98.22 (12.95) [227], 51–138	95.44 (13.63) [93], 56–128	1.71(318)	.088
CATS-Child_T0_	34.16 (8.03) [227], 21–59	34.33 (8.29) [93], 21–58	−0.17(318)	.865
CATS-Caregiver_T0_	28.70 (10.29) [219], 0–54	31.75 (8.96) [81], 14–47	−2.36(298)	.019
Treatment satisfaction (child)	3.36 (0.43) [214], 1.80–4.00	2.57 (0.85) [12], 1.30–3.70	3.22(11.32)	.008
Treatment satisfaction (caregiver)	3.59 (0.39) [196], 1.81–4.00	3.27 (0.41) [12], 2.43–3.81	2.76(206)	.006

IQ assessed with standardized tests; higher scores indicate greater cognitive ability. PTSD symptoms assessed with the CATS-2 (child- and caregiver-report); higher scores indicate greater symptom severity. Treatment satisfaction was assessed at post-treatment with the FBB scales (child- and caregiver-report); higher scores indicate greater satisfaction.

### Hypothesis 1: Relationship between IQ and PTSD symptom change

At baseline, IQ was not significantly correlated with PTSS severity in either child self-reports (*r*(324) = 0.03, *p* = 0.545) or caregiver reports (*r*(324) = −0.02, *p* = 0.743), indicating that baseline symptom burden was largely independent of cognitive ability in this sample. Regarding symptom change, bivariate correlations indicated small but significant negative associations between IQ and reductions in PTSS, both in child self-reports (ΔCATS-2_CHILD;* r*(224) = −0.14, *p* = 0.040, *n* = 226) and caregiver reports (ΔCATS-2_CARE; *r*(203) = −0.17, *p* = 0.018,* n* = 205). Higher IQ showed a small association with greater PTSS reductions. Multiple linear regressions were conducted separately for child- and caregiver-reported PTSS change, with age, gender, IQ, baseline PTSS severity (child- vs. caregiver-reported CATS-2_T0_), and treatment satisfaction assessed after treatment (child- vs. caregiver-reported FBB total mean score_T1_) entered as model variables. For child-reported PTSS change, the overall model was significant, *F*(5, 210) = 26.72, *p* < 0.001, *R*^2^ = 0.39 (adj. *R*^2^ = 0.37). IQ was not a significant predictor (β = −0.10, *t*(210) = −1.78, *p* = 0.077). Higher age was associated with smaller PTSS reductions (β = 0.18, *t*(210) = 3.04, *p* = 0.003), whereas greater reductions were related to higher baseline PTSS severity (β = −0.51,* t*(210) = −9.14, *p* < *0.001*) and higher treatment satisfaction at post-treatment (β = −0.38,* t*(210) = −6.98, *p* < *0.001*). Gender was nonsignificant (β = 0.00, *t*(210) = 0.06, *p* = 0.954). For caregiver-reported PTSS change, the overall model was also significant, *F*(5, 190) = 26.19, *p* < 0.001, *R*^2^ = 0.41 (adj. *R*^2^ = 0.39). In contrast to the child-report model, IQ emerged as a significant negative predictor (β = −0.15, *t*(190) = −2.56, *p* = 0.011), suggesting that higher IQ was associated with greater caregiver-reported PTSS improvements. Stronger PTSS reductions were related to higher baseline PTSS severity (β = −0.55, *t*(190) = *−*9.66, *p* < 0.001) and higher satisfaction ratings (β = −0.21,* t*(190) = −3.58*, p* < 0.001). Neither age (β = 0.06, *t*(190) = 1.04, *p* = 0.300) nor gender (β = 0.00, *t*(190) = −0.02, *p* = 0.987) was a significant predictor. In sum, IQ showed small significant bivariate correlations with PTSS change across both informants, but was a nonsignificant predictor in the child-report regression model and retained only a small effect in the caregiver-report model. Baseline PTSS severity and treatment satisfaction assessed after treatment each showed significant associations with symptom change across both models.

### Hypothesis 2: Relationship between IQ and dropout rate

An independent-samples *t* test showed that youth who discontinued treatment (*M* = 95.44, *SD* = 13.63, *n* = 93) did not significantly differ in IQ from those who completed treatment (*M* = 98.22, *SD* = 12.95, *n* = 227), *t*(318) = 1.71, *p* = 0.088, Cohen’s *d* = 0.21, 95% CI [−0.03, 0.45]. Consistently, the point-biserial correlation between IQ and treatment dropout was small and nonsignificant, *r*(318) = −0.10, *p* = 0.088, *n* = 320. A binary logistic regression (listwise *n* = 216) was conducted including age, gender, IQ, baseline PTSS severity (child-reported CATS-2_T0_), and treatment satisfaction at post-treatment (child-reported FBB total mean score_T1_). The overall model was significant, χ^2^(5) = 18.12, *p* = 0.003, and accounted for 24% of the variance in dropout (Nagelkerke *R*^2^ = 0.24). Model fit was acceptable, Hosmer–Lemeshow χ^2^(8) = 7.72, *p* = 0.462. IQ was not a significant predictor (Wald χ^2^(1) = 0.38, *p* = 0.536, OR = 0.99, 95% CI [0.94, 1.03]). Treatment satisfaction at post-treatment showed a strong association, Wald χ^2^(1) = 15.35, *p* < 0.001, OR = 0.13, 95% CI [0.04, 0.35], indicating that higher satisfaction was associated with reduced dropout risk. In contrast, age (Wald χ^2^(1) = 0.57, *p* = 0.450, OR = 1.09, 95% CI [0.87, 1.37]), gender (Wald χ^2^(1) = 0.00, *p* = 0.999, OR = 1.00, 95% CI [0.21, 4.75]), and baseline PTSS severity (Wald χ^2^(1) = 0.00, *p* = 0.969, OR = 1.00, 95% CI [0.91, 1.09]) were not significant. Overall, IQ was not a significant predictor of dropout in the logistic regression model, whereas satisfaction ratings at post-treatment were associated with a lower likelihood of dropout.

## Discussion

Based on theoretical models and the few available studies, we examined whether IQ was associated with PTSS reduction (Hypothesis 1) and dropout likelihood (Hypothesis 2), without specifying the direction of effects due to mixed prior findings. Overall, the results provided partial support for Hypothesis 1, with small bivariate associations, and no support for Hypothesis 2.

With respect to Hypothesis 1, bivariate analyses indicated small but significant negative associations between IQ and PTSS change in both child and caregiver reports. However, after clinical and contextual covariates were included, IQ was no longer associated with symptom change in the child-report model. In the caregiver-report model, a small effect persisted (β = −0.15), but its magnitude suggests limited clinical relevance. Overall, IQ-related effects were consistently negative but small, with higher IQ being associated with greater PTSS improvement.

These results add to the mixed and inconsistent findings reported in adults (e.g., [40, 58]) by showing that even in a large youth sample, IQ does not appear to exert a robust influence on treatment response. Notably, IQ was not associated with baseline PTSS severity in our sample, suggesting that the small bivariate correlations between IQ and symptom change were not driven by differences in initial severity. This may partly reflect range restriction in baseline PTSS due to the inclusion threshold (CATS-2 ≥ 21) and variability in the timing of IQ assessment across participants. These findings align with broader evidence that CBTs with a trauma focus produce robust effects across a wide range of patient characteristics [14], suggesting that cognitive ability may not represent a meaningful barrier to treatment benefit.

Developmental and treatment-specific features of TF-CBT may help explain this pattern. Although TF-CBT includes cognitive elements, it was developed for children and adolescents and does not rely exclusively on abstract cognitive restructuring. Rather, it combines cognitive work with psychoeducation, affect regulation, gradual exposure, skills practice, flexible trauma narrative formats, and caregiver involvement. These components can be adapted to the child’s developmental stage, language level, and emotional maturity [8]. In addition, the routine developmental tailoring inherent in child and adolescent psychotherapy may further support flexible implementation across different cognitive abilities. This structured and developmentally flexible format may reduce cognitive demands, support comprehension, skill practice, and generalization of therapeutic gains, and help account for the limited role of IQ observed in this study.

A further finding was that, across both informants, baseline severity and satisfaction ratings at post-treatment were consistently stronger correlates of symptom change, suggesting that clinical and relational factors are more relevant for PTSD recovery than cognitive ability. Because satisfaction was assessed after treatment, its association with symptom change should not be interpreted as evidence that satisfaction prospectively predicted symptom change.

The difference in the association between IQ and symptom change across child and caregiver reports may have several explanations. Children primarily report subjective distress, whereas caregivers may emphasize observable behaviors and functional impairment, which could be more loosely tied to cognitive ability. Furthermore, the heterogeneity in caregiver types in the present sample—including biological parents, foster parents, and professional or residential caregivers—may have contributed to reporting variability, as caregiver–child concordance on trauma-related symptoms has been shown to vary with the relationship to the child and the time spent together [48, 82], and professional caregivers in residential settings may have limited insight into children’s internal experiences [19, 39]. Such discrepancies between informants are well documented across child psychopathology [1, 16], with meta-analytic evidence showing that they tend to be particularly pronounced for internalizing disorders such as depression and anxiety [15], highlighting the importance of incorporating multiple perspectives in outcome evaluation.

Contrary to our assumption in Hypothesis 2, no significant association between IQ and treatment dropout was found. Dropout rates in our study (29%) exceeded meta-analytic estimates for youth trauma-focused treatments (approximately 11%; [69]). This discrepancy likely reflects the present study’s effectiveness and dissemination design, in which a clinically complex child abuse and neglect population was treated under naturalistic conditions, where broader dropout definitions and greater real-world barriers to attendance tend to inflate attrition relative to controlled trials. Higher dropout in effectiveness versus efficacy trials is a well-documented pattern [27, 83]. Critically, IQ did not contribute to this attrition risk. This stands in contrast to O’Keeffe et al. [52], who found that lower verbal intelligence predicted dropout in depressed adolescents, alongside missed sessions and poor therapeutic alliance, suggesting that the role of cognitive ability in treatment attrition may depend on the disorder, treatment modality, or population studied. Instead, perceived treatment experience was the only robust correlate of dropout in the present model, underscoring the relevance of perceived treatment experience for therapy retention. This contrasts with earlier work in women with assault-related PTSD, where lower IQ was linked to higher dropout in CPT [58], but aligns with the broader literature emphasizing that dropout is primarily driven by clinical and relational factors such as symptom severity, comorbidity, and treatment satisfaction [27, 49, 80, 88]. It should be noted that, as satisfaction was measured after treatment, the association with dropout must be interpreted cautiously. It should not be interpreted as evidence that satisfaction prospectively predicted dropout and it may reflect underlying treatment processes rather than a causal influence on attrition. Moreover, satisfaction in dropouts may have been assessed under different conditions (e.g., at discontinuation or retrospectively), which should be considered when interpreting these findings. Taken together, the role of IQ has received little empirical attention, and our findings indicate that it does not contribute meaningfully to dropout. Instead, higher satisfaction ratings were strongly associated with retention, consistent with recent evidence highlighting the importance of patient preferences [85] and family-based strategies [77] to reduce attrition.

These results extend the literature by examining IQ as an understudied predictor of treatment outcome. They further highlight the relevance of treatment satisfaction assessed at post-treatment, as it was more strongly associated with symptom change and retention than IQ, although these associations should not be interpreted as prospective effects. Finally, across both informant perspectives, TF-CBT in this dissemination trial produced large and clinically significant reductions in PTSS (Cohen’s *d* paired = 1.10–1.42). These findings are consistent with prior multicenter German studies reporting comparable and sustained TF-CBT effects in children and adolescents with abuse-related PTSD [25, 79] and support the effectiveness of TF-CBT in routine care within the observed IQ range.

### Strengths of the study

A first strength of this study is that it was conducted as a dissemination trial under real-world clinical conditions, enhancing ecological validity. Another strength is the comparably large sample size (*N* = 320) that allowed for adequately powered statistical analyses. In addition, IQ was assessed using validated, age-appropriate standardized instruments rather than proxy indicators such as educational attainment, which represents an important methodological advantage over prior work [17, 43]. The observed IQ range was broad (51–138), allowing us to examine associations across a relatively wide range of clinically assessed cognitive functioning. Finally, PTSD symptoms were assessed from multiple informant perspectives (youth and caregivers), and the multi-informant design increases confidence in the reliability and validity of the outcome assessment [62].

### Limitations and future research directions

Despite these strengths, several limitations should be noted. The naturalistic design of this dissemination trial may have reduced internal validity due to uncontrolled factors such as therapist variability or contextual differences across treatment sites. In addition, treatment duration was not standardized and varied across participants, which could have influenced outcomes, as intelligence may relate differently to short- versus long-term interventions [32]. Moreover, therapist adherence to the TF-CBT manual was assessed in the larger study but was not included in the present analyses, which limits conclusions. Unmeasured confounders—particularly socioeconomic status (SES), which is closely linked to both IQ and mental health—may also have influenced outcomes [20]. Future work should therefore include systematic assessment and statistical control of SES to better isolate the role of cognitive ability. As IQ was not experimentally manipulated, causal relationships between cognitive ability and treatment outcome cannot be inferred, and bidirectional effects cannot be ruled out.

A further important limitation concerns the heterogeneous assessment of IQ across sites. Although all IQ scores were based on standardized and age-appropriate instruments, the specific intelligence test used varied across sites and participants. The measure selected depended on local clinical routines, participant age, available prior standardized assessments, and available assessment resources. This approach is consistent with the naturalistic character of the dissemination trial and reflects routine clinical practice, where diagnostic procedures must often balance methodological standardization with clinical feasibility, available assessment instruments, clinical time, and resource constraints. At the same time, this represents a substantial methodological limitation. Different intelligence tests may place different emphasis on specific cognitive domains, such as verbal comprehension, fluid reasoning, working memory, processing speed, or nonverbal reasoning. As a result, the IQ variable used in the present analyses cannot be interpreted as a uniform measure of a specific component of intelligence. Rather, the findings should be interpreted as referring to broad clinically assessed IQ scores derived from different standardized instruments and cannot clarify whether particular cognitive domains are more or less relevant for TF-CBT outcomes.

Assessments were also carried out by treating therapists rather than uniformly trained research diagnosticians, which may have introduced additional variability or rater bias. Future studies should therefore use a uniform comprehensive IQ instrument across sites, ensure consistent assessor training, or include domain-specific cognitive measures to examine whether particular cognitive abilities are differentially associated with trauma-focused treatment response.

Another limitation concerns the limited representation of youth with intellectual disabilities. Although the observed IQ range extended down to 51, youth with IQ scores below 50 were excluded, and the sample included relatively few participants in the lower intellectual functioning range. Consequently, the findings should not be generalized uncritically to youth with moderate or severe intellectual disability. This limitation is clinically important because children and adolescents with intellectual disabilities are at elevated risk of victimization and post-abuse mental health problems, yet they remain underrepresented in trauma treatment research [10]. This underrepresentation is also evident in clinical trial eligibility criteria, as Moore et al. [50] found that 61% of child PTSD treatment RCTs in current clinical care guidelines applied exclusion criteria similar to those used in the present study. Initial clinical guidance for adapting TF-CBT for this population has been proposed [26], but empirical evaluation remains needed. However, broader evidence from trauma-focused treatment research supports the potential value of cognitively tailored approaches. For example, Crocker et al. [9] demonstrated that modifying CPT to accommodate executive functioning deficits improved outcomes in veterans with PTSD and traumatic brain injury. Future studies should therefore specifically examine adapted TF-CBT protocols for youth with intellectual disabilities and investigate whether and which modifications are needed to ensure accessibility and clinical benefit.

Furthermore, IQ was not uniformly assessed at a single time point: some scores were obtained from prior clinical assessments conducted by other clinicians, while others were collected at baseline by treating therapists. As a result, the temporal relationship between trauma exposure and cognitive testing varied across participants, and measured IQ scores may in some cases partly reflect trauma-related cognitive impairment rather than stable premorbid ability [21, 30, 31]. This potential confound may attenuate the observed association between IQ and treatment response and limits causal inferences about the role of cognitive ability in TF-CBT outcomes. In addition, participation was voluntary, so selection effects cannot be excluded; for instance, families with higher motivation or fewer cognitive barriers may have been more likely to complete treatment. Another limitation concerns dropout, which was not explored through systematic follow-up with patients, caregivers, or therapists. This limits insight into the underlying reasons for premature termination. Incorporating qualitative methods in future research (e.g., interviews) would provide a more nuanced understanding of dropout dynamics [29, 73]. Finally, caregiver data were available only for smaller subsamples and included substantial missing data, which constrains the interpretation of caregiver perspectives. Given the central role of caregivers in TF-CBT for children and adolescents [7, 8], future studies should examine more systematically how caregiver characteristics—including their cognitive abilities, engagement, and support—shape treatment outcomes.

### Clinical implications

From a clinical perspective, the findings suggest that higher cognitive ability is not a prerequisite for benefiting from TF-CBT in youth with PTSD. Within the observed IQ range, TF-CBT proved effective under routine care conditions.

Because children with severe intellectual disability (IQ < 50) were excluded, our findings cannot be generalized to this group. Nevertheless, the results provide reassurance that TF-CBT can be successfully applied across a broad range of cognitive abilities. Prior work has emphasized that TF-CBT is most effective when adapted to individual needs and delivered in a supportive manner [8, 25]. Although our data suggest that lower IQ within the tested range was not a major barrier to treatment benefit, previous work indicates that flexible adjustments—such as simplifying language, using visual supports, or enhancing caregiver involvement—may help optimize accessibility and support comprehension and generalization of therapeutic content in diverse youth populations [13, 56]. Hoover et al. [26] have proposed a systematic framework for tailoring individual TF-CBT components to children with mild-to-moderate intellectual disabilities, organized around key functional domains including comprehension, executive functions, and generalization, which may inform future adaptations for lower-functioning youth.

The robust and clinically meaningful effects observed in this dissemination trial strengthen confidence in TF-CBT as a first-line intervention for traumatized youth. These findings support its broad implementation in routine care without pre-screening for cognitive ability, thereby lowering barriers to treatment and facilitating large-scale roll-out.

Finally, these findings support the use of TF-CBT in routine care across the observed range of cognitive ability levels while also highlighting treatment satisfaction as an important aspect of patient experience. At the same time, the limited representation of youth with intellectual disabilities underscores the need for future research to examine accessibility and treatment benefit in this group more directly.

## Data Availability

The datasets generated and/or analysed during the current study are not publicly available due to privacy and ethical restrictions related to patient confidentiality. Data may be made available from the corresponding author on reasonable request, subject to institutional approval and applicable data protection regulations.

## Abbreviations

CAN: Child abuse and neglect
CATS-2: Child and adolescent trauma screen, version 2
CPT: Cognitive processing therapy
DSM-5: Diagnostic and statistical manual of mental disorders, fifth Edition
FBB: Fragebogen zur Beurteilung der Behandlung
FBB-E: Fragebogen zur Beurteilung der Behandlung—Elternversion (caregiver)
FBB-KJ: Fragebogen zur Beurteilung der Behandlung—Kinder/Jugendliche
ICD-10: International classification of diseases, 10th revision
ICD-11: International classification of diseases, 11th revision
IQ: Intelligence quotient
PTSD: Posttraumatic stress disorder
PTSS: Posttraumatic stress symptoms
RCT: Randomized controlled trial
SAU: Supervision as usual
SES: Socioeconomic status
TF-CBT: Trauma-focused cognitive behavioral therapy
TFS: Trauma-focused supervision
